# Virtual/augmented reality-based human–machine interface and interaction modes in airport control towers

**DOI:** 10.1038/s41598-024-63731-3

**Published:** 2024-06-12

**Authors:** Sara Bagassi, Marzia Corsi, Francesca De Crescenzio, Ramona Santarelli, Aurora Simonetti, Laura Moens, Michela Terenzi

**Affiliations:** 1 Department of Industrial Engineering, University of Bologna, 47121 Forlì, Italy; 2 ENAV S.p.A., 00138 Rome, Italy; 3 Deep Blue, 00185 Rome, Italy

**Keywords:** Air traffic control, Human machine interface, Augmented reality, Multimodal interaction, Airport control tower, Safety nets, Aerospace engineering, Electrical and electronic engineering

## Abstract

The concept of an innovative human–machine interface and interaction modes based on virtual and augmented reality technologies for airport control towers has been developed with the aim of increasing the human performances and situational awareness of air traffic control operators. By presenting digital information through see-through head-mounted displays superimposed over the out-of-the-tower view, the proposed interface should stimulate controllers to operate in a head-up position and, therefore, reduce the number of switches between a head-up and a head-down position even in low visibility conditions. This paper introduces the developed interface and describes the exercises conducted to validate the technical solutions developed, focusing on the simulation platform and exploited technologies, to demonstrate how virtual and augmented reality, along with additional features such as adaptive human–machine interface, multimodal interaction and attention guidance, enable a more natural and effective interaction in the control tower. The results of the human-in-the-loop real-time validation exercises show that the prototype concept is feasible from both an operational and technical perspective, the solution proves to support the air traffic controllers in working in a head-up position more than head-down even with low-visibility operational scenarios, and to lower the time to react in critical or alerting situations with a positive impact on the human performances of the user. While showcasing promising results, this study also identifies certain limitations and opportunities for refinement, aimed at further optimising the efficacy and usability of the proposed interface.

## Introduction

In conventional and remote airport control towers, air traffic controllers (ATCO) provide airport control service using human sight and are supported by different systems. Each one of these auxiliary tools, such as surveillance radars (ground and air), Meteorological Aerodrome Report, and flight data processing, requires at least one screen or human–machine interface, forcing the controllers to increase the time spent looking down at the screens^1^ and continuously switch between a head-down (looking at the auxiliary tools) and head-up (looking Out of The Window—OTW) position. According to human factor research^2–4^ not only could the continuous change of perspective of the same environment lead to a decrease of the situational awareness, but the time spent in a head-down position should be reduced to lower the risk of not detecting unpredictable situations^5,6^. With the aim of increasing direct head-up observations of the OTW and supporting Air Traffic Control Operations, several preliminary studies and projects have been carried out over the last two decades thanks to the advancement of technologies such as Virtual Reality (VR) and Augmented Reality (AR) and remote and virtual control towers (RVT)^7,8^.

As a matter of fact, in an airport control tower environment, Extended Reality could be used to display additional auxiliary computer-generated visual information as an overlay over the real-world data and blended with the out-of-the-window view to improve identification and tracking of aircraft especially in low visibility conditions. Moreover, with the introduction of these technologies, ATCOs attention would not constantly be divided between two different perspectives of the same environment (primary out-of-the-tower visual field and auxiliary head-down equipment) with a benefit in terms of increased Situational Awareness (SA) and reduced workload.

Reisman^9^ developed and tested a first set of possible solutions for the provision of Augmented Reality in 2006 . The validation campaign reported several issues resulting from the immaturity of the proposed technologies, still recognising the great potential of AR in the provision of the needed surveillance information to tower operators. In 2007, Tavanti^10^ highlighted the need to include users in the design of AR tower applications, given the high level of specificity of any traffic scenario. Given a big step in the technology maturation, a second wave of research activity started in the mid-2010s. Silva et al.^11^ compared and integrated multiple surveillance sources (radar and ADS-B) to point the aircraft position on a video-based AR interface in 2015. Gürlük^12^ in 2016 evaluated the beneficial effects of AR in increasing situational awareness, whether recognising some critical aspects of the ergonomics of the devices and the data organisation and visualisation in a head-mounted display (HMD). Whilst, in 2018, Gürlük et al.^13^ tested a first implementation of AR in a virtual traffic scenario simulation involving tower controllers. The results showed increased task performance and overconfidence, which was partially due to AR.

**Figure 1:**
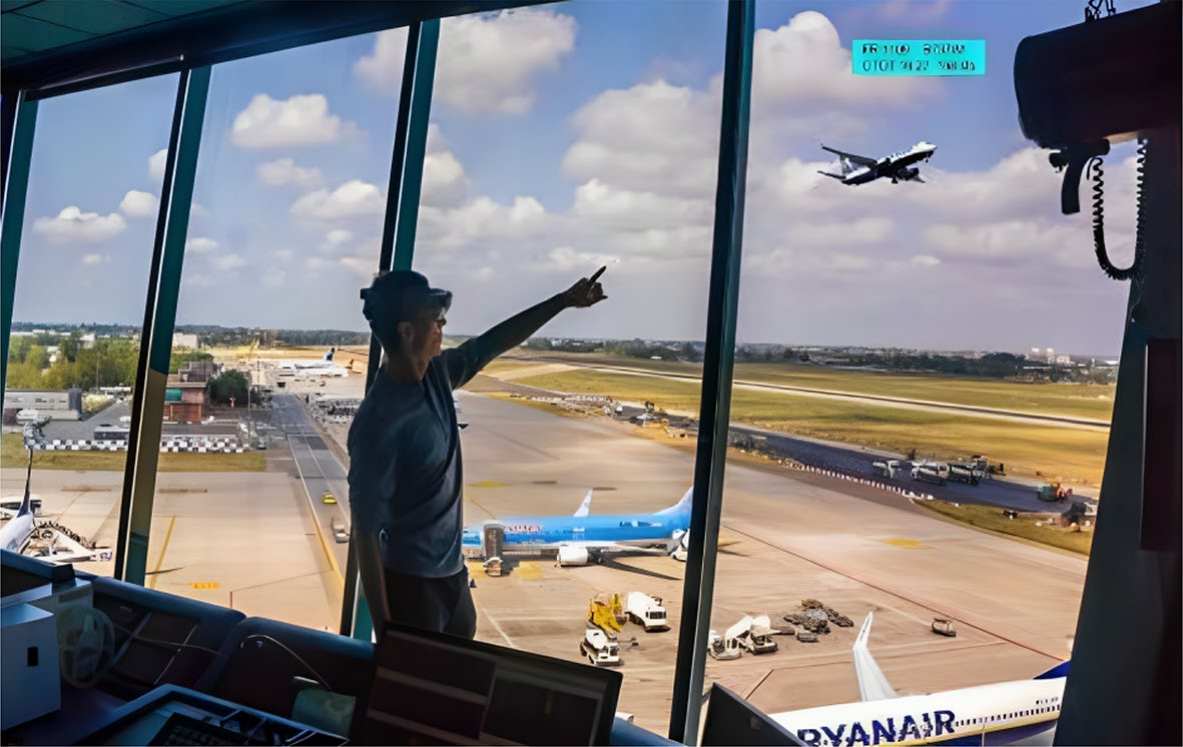
*RETINA* concept—an operator with the HMD and the displayed information seen superimposed through the window.

At the same time, in 2016, Bagassi et al. proposed the *RETINA* concept^14^ (Fig. 1). The *Resilient Synthetic Vision for Advanced Control Tower Air Navigation Service Provision - RETINA* project^15^ has been one of the first selected Single European Sky ATM Research (*SESAR*) projects on High Performing Airport Operations aimed at the investigation of the potential and applicability of Virtual/Augmented Reality technologies for the provision of Air Traffic Control service by the airport control tower^16,17^. The idea behind *RETINA* exploratory research was to overlay additional synthetic information such as flight tags, aerodrome layout and Meteorological Aerodrome Report information over the actual out-of-the-tower view through conformal-head-up displays (C-HUD) or see-through head-mounted displays (ST-HMD).

As expected, enabling the controllers to have a head-up view of the traffic even in bad visibility conditions proved to be beneficial for the ATCOs’ human performance while maintaining safety and increasing airport resiliency in low visibility^18^. Moreover, the results obtained by the *RETINA* consortium, coordinated by the University of Bologna, served as a basis for the *SESAR JU Digital Technologies for Tower (PJ05-W2 DTT)* project^19,20^.

The *DTT* project aims to contribute to Air Traffic Management (ATM) digitalisation objectives both by enhancing and developing the concept of innovative AR-based human-machine interface (HMI) and interaction modes in airport control towers, and by proposing the development of a remote aerodrome air traffic service where services from various aerodromes are combined in a centralised control room independent of airport location. The project is composed of three different solutions, each of which focuses on different purposes to be validated and progressively developed for the benefit of the ATM network in terms of safety, capacity, efficiency and flexibility. The first solution, *Multiple Remote Tower and Remote Tower Centre*, focuses on the remote provision of Air Traffic Services from a Remote Tower Centre (RTC) to a number of airports including the development of RTC supervisor and support systems. Advanced automation functions, integration of approach for airports connected to the remote centre and connections between RTCs with systems for flow management are covered by the solution together with the development of tools and features for a flexible planning of the aerodromes connected to the remote tower services. The second and third solutions, *ASR at the TWR CWP supported by AI and Machine Learning* and *Virtual/Augmented Reality applications for Tower*, deal with both the current operating airport environment and future environments. The core of the activities is oriented towards the following two main areas: Automatic speech recognition (ASR) and Virtual and Augmented Reality. VR/AR in different applications allows the controllers to conduct safe operations under any meteorological conditions while maintaining a high taxiway and runway throughput. Within this area other technologies such as tracking labels and air gestures and attention guidance are investigated.

For this purpose within in the framework of the *Virtual/Augmented Reality applications for Tower* solution, the project partners planned three different exercises on three different simulation scenarios assessing different aspects of Tracking Labels, Multimodal interaction and Attention Guidance.

- Validation of AR Interaction Modes for Schiphol Tower with a Focus on Attention Guidance—conducted by the Koninklijke NLR—Nederlands Lucht—en Ruimtevaartcentrum
  Carried out as a real-time simulation to address the use of attention capturing and guidance as new interaction modes for controllers in a customized environment representing the aerodrome control tower at Amsterdam Schiphol Airport (EHAM)^21^.
- Augmented Reality Multimodal Control Tower Interaction—conducted by ENAV S.p.A., University of Bologna and DeepBlue.
  A real-time simulation addressing Virtual/Augmented Reality Tower Tools, Tracking Labels, Air Gesture Interaction and Safety Nets at Bologna Airport (LIPE).
- Augmented Reality in the Tower Environment—conducted by ENAIRE and CRIDA.
  Shadow mode validation exercise to address Augmented Reality, Tracking Labels and Air Gestures at Vitoria-Gasteiz Airport (LEVT)^22^.

This paper targets and describes the *Augmented Reality Multimodal Control Tower Interaction* Bologna validation exercise by focusing on the simulation platform and exploited functions to evaluate whether and how VR/AR along with Tracking Labels, Air Gestures and Safety Nets can allow ATCOs to increase head-up time, even in low visibility conditions, to lower the time to react to critical or alerting situations, to reduce the workload, and to improve SA and productivity.

## Digital technologies and human–machine interaction in airport control towers

The concept of an augmented reality human–machine interface (AR HMI) for airport control towers was initially explored in the *RETINA* research project. Further investigation into this concept is underway within the *SESAR JU Digital Technologies for Towers* (*DTT*) industrial research project.

As part of the *DTT* project, a specific focus was given to the development of the solution *Virtual/Augmented Reality Applications for Tower Operations*, building upon the findings of the *RETINA* project^18^. The objective of the solution’s exercise *Augmented Reality Multimodal Control Tower Interaction* was to refine and expand upon the findings of the *RETINA* validation campaign, addressing several identified research gaps. These included the need for multi-user operations, understanding the impact of the shift in visibility conditions on ATCOs tasks, and exploring various interaction options, including multimodal interaction.

This paper presents the integration of additional features not previously considered at the exploratory research stage. These include (a) adaptive Human–Machine Interface (HMI) and working positions, (b) multimodal interaction, and (c) safety nets visualization.

The exercise aimed to achieve several outcomes:

- Providing consistent visual conditions for tower controllers in all weather conditions through the use of Virtual and Augmented Reality technologies with head-up interfaces.
- Enhancing controller productivity and situational awareness by minimizing the need to switch between head-up and head-down positions.
- Facilitating more natural and efficient interaction in tower control operations through the implementation of air gestures.
- Improving safety by displaying perceptual cues that direct controllers’ attention to specific events.

To validate these hypotheses and address the gaps identified by *RETINA*, a validation campaign was conducted using the simulated scenarios of Bologna International Airport (LIPE), which had previously been utilised during the *RETINA* campaign.

Bologna airport is equipped with Primary and Secondary Surveillance RADAR, Surface Movement Radar and Instrumental Landing System CAT 3B, it has a moderately complex layout (one runway, several taxiway, more than one apron) with moderate traffic (between 200 and 300 movements per day). Moreover, Bologna is a single runway (12 and 30) airport with a main taxiway T and several taxiway and aircraft stand taxilanes, the runway has a 12/30 orientation with an asphalt strip of 2803x45 m. Aircraft may only take off from, and land on Runway 12 when Low Visibility Procedures (LVP) apply.

LVP are international operating procedures adopted during low visibility conditions to enable aircraft to take off and land in complete safety, and in Bologna airport are available in accordance with three visibility conditions. Visibility condition 1 (CONDI VIS 1) means normal operations since it is considered when the visibility is sufficient for the pilot to taxi and to avoid collision with other traffic on taxiways and at intersections by visual reference, and for personnel of control units to exercise control over all traffic on the basis of visual surveillance. In visibility condition 2 (CONDI VIS 2) the manoeuvring area is not completely visible from the control tower, therefore it corresponds to the instrument meteorological conditions (IMC) scenario in which visibility is sufficient for pilots to taxi and to avoid collision with other traffic on taxiways and at intersections by visual reference, but insufficient for personnel of control units to exercise control over all traffic based on visual surveillance. Visibility condition 3 (CONDI VIS 3) implies a runway visibility range lower than 400 m but higher than 75 m. It is an IMC scenario in which visibility is sufficient for pilots to taxi but insufficient for the pilot to avoid collision with other traffic on taxiways and at intersections by visual reference, and insufficient for personnel of control units to exercise control over all traffic based on visual surveillance. Not only does a drop in visibility conditions lead to more stringent operational rules in terms of separation between aircraft and number of flights managed, but the working method of the ATCOs also has to change relying more on the auxiliary tools and therefore increasing head-down operations.

**Figure 2:**
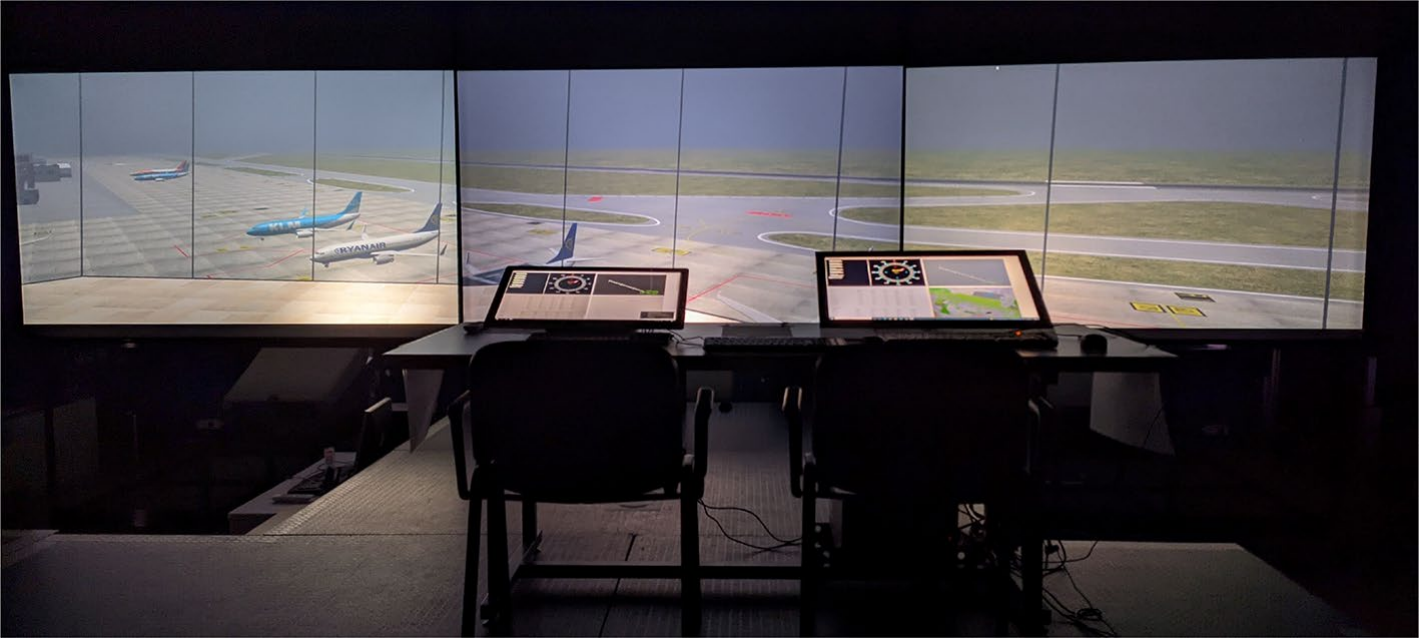
Validation platform implemented in the CAVE-like virtual environment of the University of Bologna.

In order to investigate the use of VR/AR in a conventional control tower environment at Bologna airport with a specific focus on adaptive HMI, working position, multimodal interaction, and safety net visualisation, a validation campaign was performed in a semi- immersive CAVE-like virtual environment (Fig. 2).

The campaign comprised three different validation exercises assessing the technical solution, see-through head mounted smart glasses for the viewing of holograms that are integrated within a real world environment, with three different associated functions: Tracking Labels (TL), Air Gesture (AG) interaction and Safety Nets (SN).

For each feature, the technical solution which supports the controllers by providing information as overlays over the external view, was evaluated against baseline equipment. The** reference scenario (baseline) **is a replica of the out-of-the-tower view of the Bologna aerodrome including a simplified head-down display interface comprising weather information, Flight Duty Period, Approach Radar and Ground Radar.

**Figure 3:**
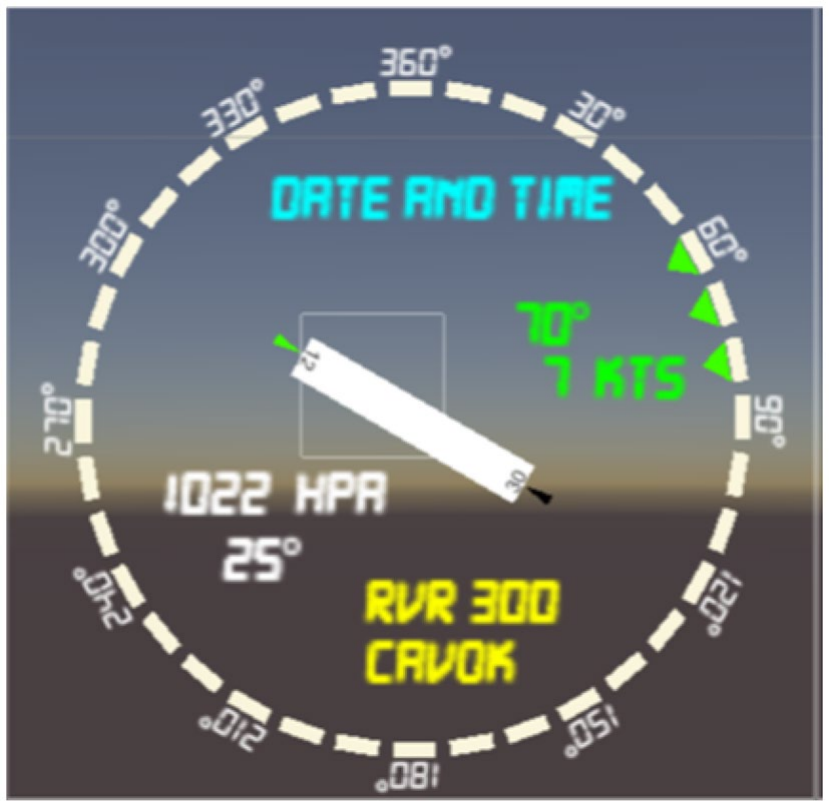
Meteorological aerodrome report (METAR) interface.

The **solution scenario** adds to the out-of-the-tower view and baseline head-down equipment overlays of digital data tailored according to user operative position, gaze orientation, phase of flight and visibility condition. The digital information shown is Meteorological Aerodrome Report (METAR), tracking labels with aircraft identification and status, and airport layout overlays. The METAR interface (Fig. 3), reports date and time, runway in use, the wind direction and intensity, pressure and temperature on the runway and the runway visibility range (RVR).

**Figure 4:**
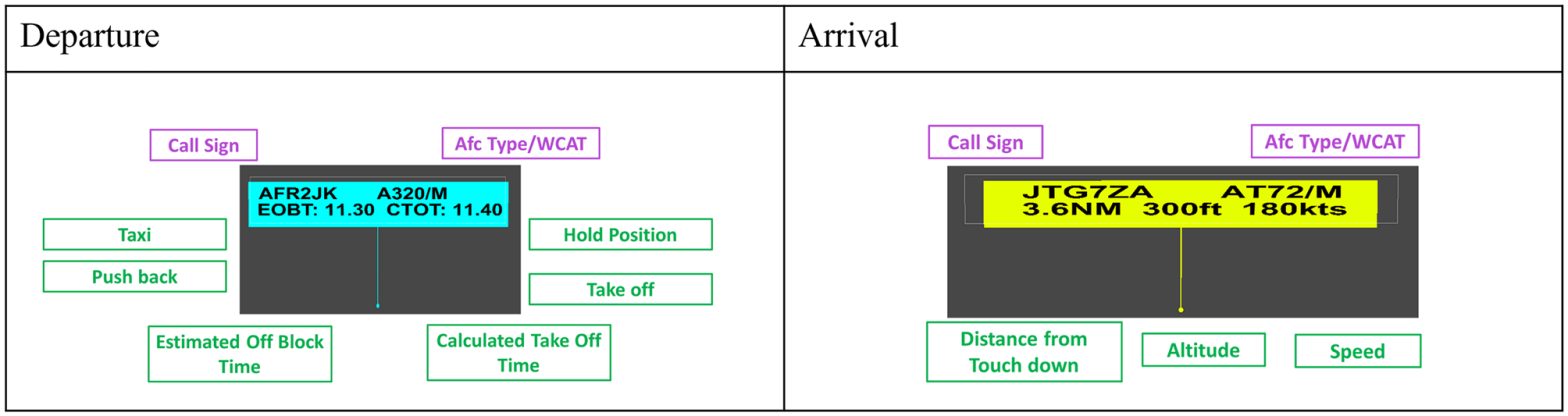
Aircraft tracking labels, permanent information is reported on the first line of the label, adaptive information on the second one.

As can be seen in Fig. 4, each tracking label is linked to the associated aircraft with a bar and can have two different colours, cyan for departures and yellow for arrivals. The information reported on the tracking labels is of two types, permanent (Call Sign and Afc Type/WCAT) and adaptive (EOBT, CTOT, Push Back, Taxi, Hold position, Take off for departure label and Distance from touch down, Altitude and Speed for arrival label).

**Figure 5:**
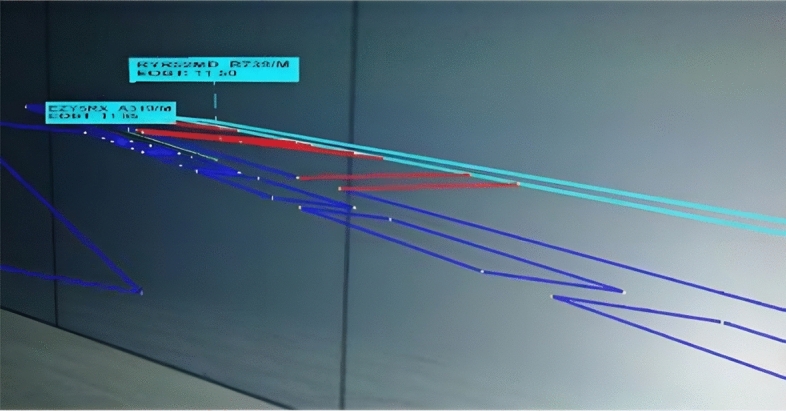
Aircraft tracking labels and airport layout overlays in low visibility conditions (CONDI VIS 3), the colour of the runway follows the same coding of the aircraft TL.

Moreover, as the visibility condition decreases, airport layout overlays appear as can be seen in Fig. 5. Whilst the taxiway colour is always blue, the colour of the runway changes according to the phase of flight of the aircraft which is occupying it, cyan if an aircraft is taking off and yellow if it is landing.

**Figure 6:**
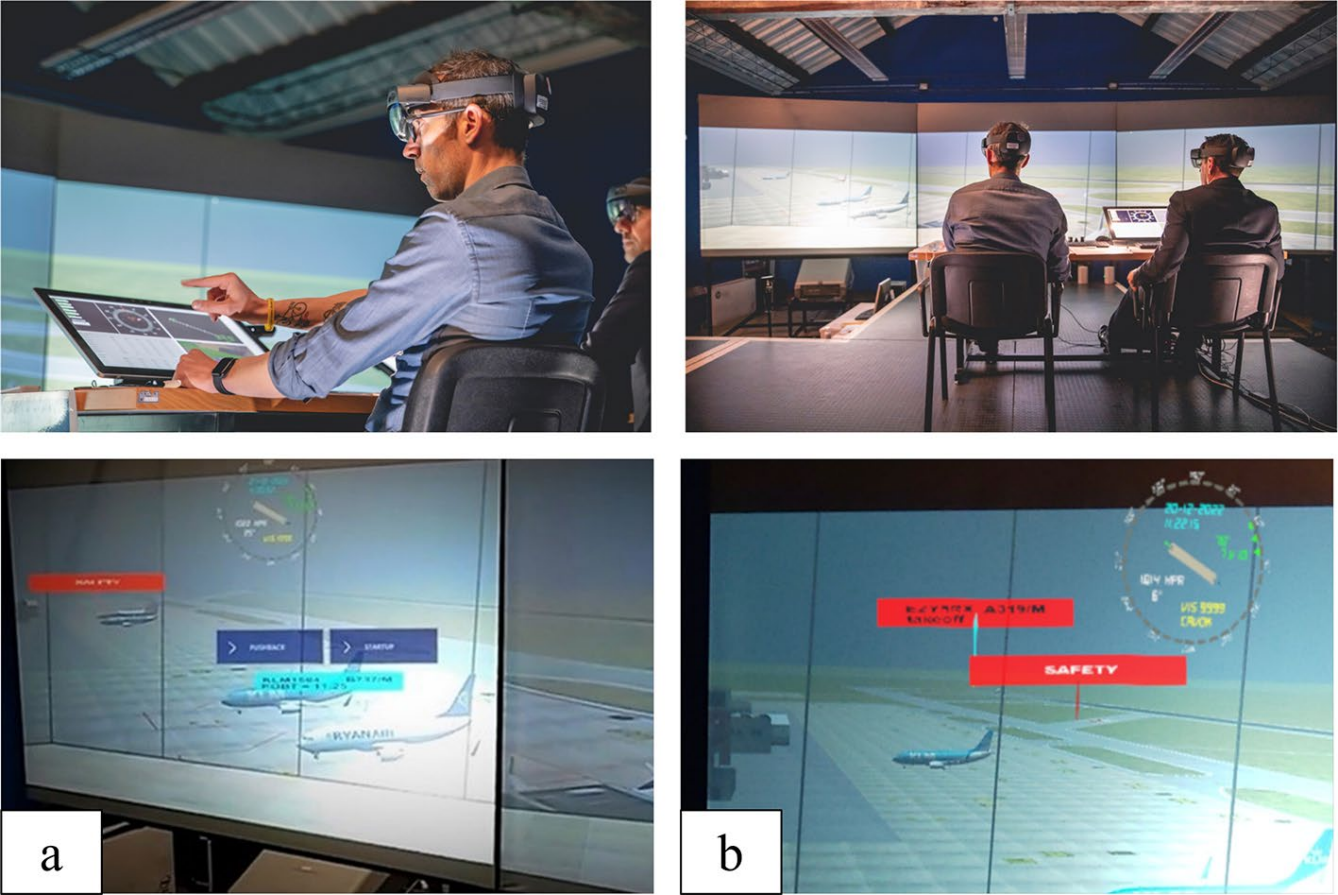
ATCOs can simultaneously see both the out of the tower view and the AR overlays through the head mounted smart glasses (Microsoft HoloLens2). (**a**) Personal view of the GND controller during the Air Gestures solution, the blue buttons allow the ATCO to issue *Push back* and *Start-up* clearances to the pseudo-pilot. (**b**) Personal view of the RWY controller during the Safety Net solution exercise.

By presenting digital data, virtual and augmented reality along with tracking labels and air gestures are expected to give the controller the possibility of an increased head-up time of the airport traffic, even in low visibility conditions. Furthermore, in good visibility some of the limitations regarding the display of information (e.g. planning times and warnings) that might be missed due to increased focus on the outside view, can be mitigated. In addition, safety nets can support the controller in reacting to critical situations when and where needed. By means of this solution, the controllers will no longer be limited by what the human eye can physically see out of the tower windows (Fig. 6), and consequently will be stimulated to operate in a head-up position and reduce the number of head-up/head-down switches. This is expected to lead to an increased ATCO situational awareness, increase of controller’s productivity and a reduction in reaction times.

## Methods

This experimental campaign was conducted following Horizon 2020 ethics guideline and as requested by the European Union’s grant agreement No. 874470. The experimental data were collected anonymously in the form of observations, self-assessment questionnaire and non-invasive, objective measurement techniques with no associated risk.

The ethical dimension of the project was assessed using Horizon 2020 Ethics Appraisal Procedure. Based on the ethics assessment performed by Horizon 2020, no ethics issues were identified. However, the experimental protocol was approved by the Project Management Board composed of the following institutions: Austro Control, Croatia Control, DLR - Deutsches Zentrum für Luft- und Raumfahrt, ENAV, University of Bologna.

In order to participate to the study, all the participants (recruited on a voluntary basis) and the institution to which they belong provided informed consent and permitted the release of identifying images for collection and open-access publication.

### Apparatus design

In order to validate the concept developed, a Human-in-the-loop real-time simulation was planned at the Virtual and Simulation Laboratory of the University of Bologna simulation and validation platform. The platform comprehends a multipurpose CAVE-like virtual environment and starting from 2016 has been customized as a Control Tower Simulator including controller working position and pseudo-pilot posts, to perform research on newly conceived HMI for Airport Control Towers. The platform is able to replicate any Airport environment and out-of-the-tower view, and to simulate different visibility conditions. For the specific purpose of the presented study, aiming at maturing the results obtained during the *SESAR* exploratory research project *RETINA*, the validation platform, was based on *RETINA* experimental campaign’s platform and Bologna Airport (LIPE) was selected as operational scenario. For further understanding of the *RETINA* platform and validation campaign, the reader is referred to^18^. Moreover, additional components and devices were integrated to the platform to assess the three following components under investigation.

**Adaptive HMI and CWP:** the simulation involves two different controller working positions (CWP), namely Tower Ground and Tower Runway. To fully customise the type of information delivered and the view of each one of the two users, the system has to track two different points of view.

**Multimodal interaction:** the users are enabled to interact with the system by a combination of voice and air gestures. In this specific campaign, datalink-like messages related to not-time-critical clearances (Push back and Start-up) can be issued by means of multimodal interaction.

**Safety net:** safety warnings regarding conflicting clearances and runway incursions are displayed through VR/AR overlays and directional sound alarms guide the attention of the ATCOs towards critical events.

**Figure 7:**
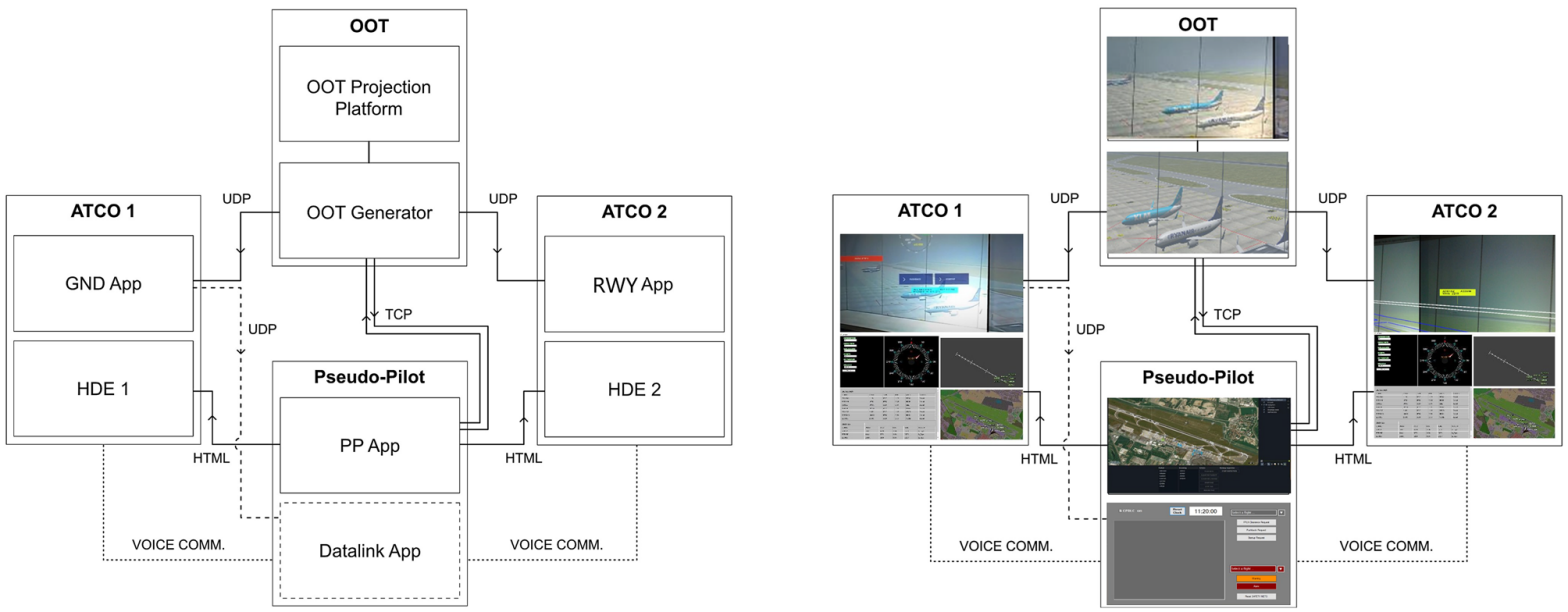
The validation platform consists of two air traffic controller posts that communicate to a pseudo-pilot post. The platform can simulate any airport environment in different visibility conditions by means of a full 4D model system exchanging data with five subsystems: out of the tower view generator (OOT), ground augmented reality overlay application (GND App), runway augmented reality overlay application (RWY App), head down equipment (HDE) and pseudo-pilot application (PP App).

The exercise platform architecture consists in five modules feeding three different role’s posts (ATCO GND, ATCO RWY and Pseudo-pilot) as depicted in Fig. 7.

The core system of the platform is a 4D model of the reference scenario which integrates the data sources, responds to user inputs and manages events. This module is also in charge of the communication with five subsystems:

- **Out of the tower view generator (OOT):** the OOT provides the ATCOs with a realistic and consistent scenario of the out-of-the-tower view of the aerodrome with a CAVE-like virtual environment. The platform can simulate any airport environment in different visibility conditions. Bologna (LIPE) aerodrome and different visibility conditions were selected for the presented simulation campaign.
- **Ground augmented reality overlay application (GND App) and runway augmented reality overlay application (RWY App):** the augmented reality overlay applications (GND App and RWY App) are tailored with respect to the specific working position in terms of both point of view and necessary information (e.g. visibility conditions and flight status), and derive and deploy the AR overlays on two Microsoft HoloLens2 head mounted see-through displays. This module is only activated for in solution scenario.
- **Head down equipment (HDE):** the CWP includes an HDE presenting a set of data similar to what is given to the ATCOs via the actual head-down equipment in the control tower. It derives data from the 4D model and presents them to the ATCO on a screen. Each working position is equipped with one HDE provided onto a 27” display.
- **Pseudo-pilot application (PP App):** through the PP App the pseudo-pilot is enabled to monitor and update the state of the 4D model module according to the controllers’ instructions. The pseudo pilot post includes an additional interface for Controller-pilot datalink communications (CPDLC). This interface is used for the scenarios related to Air Gestures and Safety Net to allow the user to send specific clearance requests and to receive datalink-like messages from the ATCO and to reset the alarm related to the runway incursion.

### Objectives and metrics

The validation objective was the assessment of the introduction, at different levels of maturity, of VR/AR technologies in airport control towers according to the following key performance areas (KPAs): human performance and safety. After obtaining the informed consent from all the participants to the validation tests, different metrics were collected anonymously in the form of:

- Objective quantitative measurements recorded by the platform for post-run analysis, namely: head-up time and number of switches head-up/head-down, number of vocal communications and time to react to safety events.
- Subjective qualitative assessments such as workload, acceptability, trust, usability, human error and user comfort obtained through questionnaires and/or interviews.

Standard scale questionnaires were employed to measure controllers workload, situational awareness and acceptance: Bedford^23^, China Lake^24^ and CARS^25^ scale were administered respectively after each exercise runs (for both reference and solution runs). Customised questionnaires were employed post runs to collect further Human Performance and Safety subjective measurements, and a post-experiment questionnaire to collect a final subjective overview of the new solutions proposed.

The selected tools for assessing human performances were chosen from commonly used ones in *SESAR* framework to assess ATCOs workload and performances. For the air gesture solution, due to the lower level of target maturity, only structured debriefings were conducted to collect initial subjective feedback on the use of the technology.

### Participants and test execution

**Figure 8:**
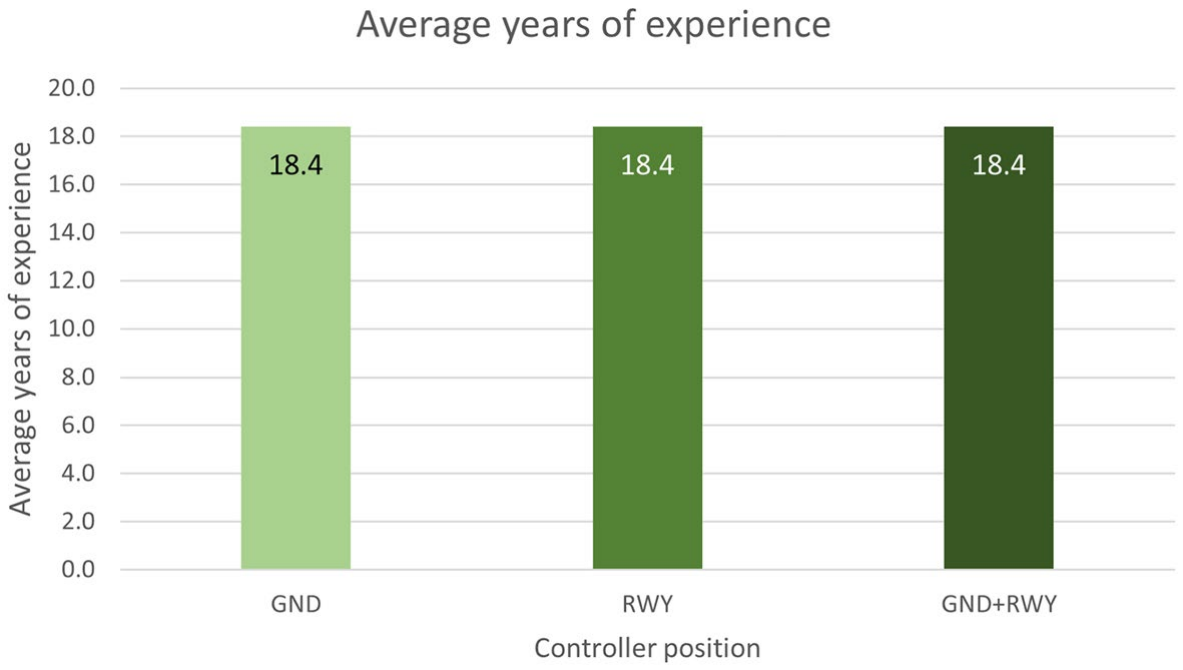
Average years of experience of the ATCOs involved in the campaign.

**Figure 9:**
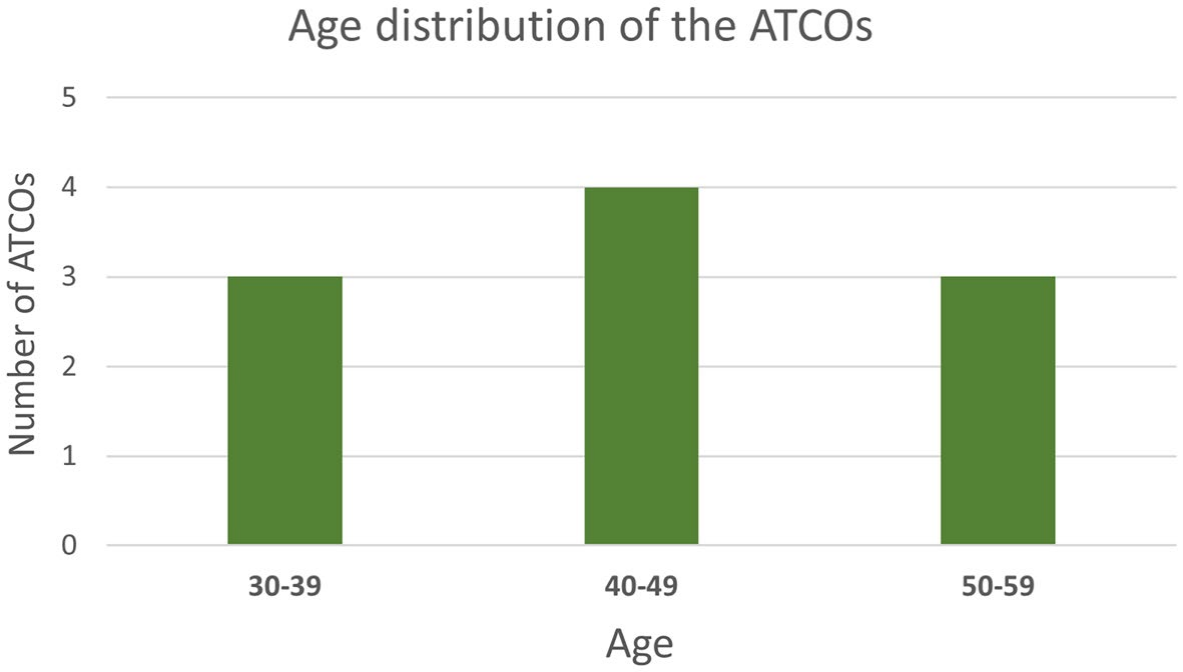
Average age distribution of the ATCOs involved in the campaign.

During the Validation exercise, a total of ten experienced ATCOs divided into five groups took part in a Human-in-the-loop (HITL) Real-time Simulation (RTS) in a scenario replicating the Bologna aerodrome. In each one of the five teams involved in the simulations one controller was assigned to the GND and one to the RWY position with no rotation of the users among the two CWPs. Data regarding the age and the years of experience of the controllers are shown in Table 1 and in Figs. 8 - 9.

**Table 1:** Average years of experience along with standard deviation (in brackets) of the ATCOs involved in the campaign.

	GND+RWY	GND ATCO	RWY ATCO
Average years of experience (SD)	18.4 (7.6)	18.4 (4.8)	18.4 (9.6)

The ground controller was responsible for providing the aerodrome control service and the flight information service on the manoeuvring area except the runway. The runway controller was responsible for providing the same services in the aerodrome traffic zone and on the runway. In order to assess the introduction of the specific feature, namely tracking labels, air gestures and safety nets, each simulation scenario in which the GND and RWY ATCOs were supported by the technical solution was preceded by a reference with same amount of traffic and visibility conditions which was conducted using only adopting the baseline equipment (HDE), see Table 2. Given the total of five controllers in each position, a thorough evaluation of the concept for different aspects and from different perspective was ensured.

As stated before, the validation exercise addressed three different features.

- Tracking Labels
  The simulation involving the use of VR/AR Tracking Labels is performed on a 40 min solution scenario including 11 movements (4 arrivals and 7 departures). The VR/AR overlays are displayed through Microsoft HoloLens2 head-mounted display and are tailored according to the user’s working position and point of view, as well as the specific visibility conditions. The visibility conditions gradually degrade over the course of the exercise Run with 15 min of CONDI VIS 1, 10 min of CONDI VIS 2 and 15 min of CONDI VIS 3. As the low visibility conditions become more severe, additional airport layout overlays are displayed as reported in Fig. 5.
- Air Gestures
  Given the lower maturity level of the solution related to Air Gestures, the associated validation run is performed on a 15 minute exercise in good visibility conditions (CONDI VIS 1). The AR application enables the GND controller to interact with the AR overlays through the use of the gestures currently recognised by the HoloLens2 device (see Fig. 6) to manage not-time-critical tasks such as Departure, Start-up and Push back clearances release.
- Safety Nets
  To assess the benefit of the introduction of Safety Nets to guide the attention of the ATCOs, a technical test is executed on a 30 minute exercise in good visibility conditions. A safety event is simulated when the pseudo-pilot starts an unauthorised runway inspection with the runway occupied by an aircraft. The event triggers the activation of a red tracking label for the specific aircraft, and a directional acoustic alarm sounds to drive the attention of the controller in the right direction and to compensate for the reduced augmented field-of-view of the HMD device.

**Table 2:** The experimental plan consists of five exercises (Run) performed in two scenarios.

	Reference	Solution
Scenario 1 40’ visibility variation	Run 1	Run 2 (VAR + TKL)
		Run 3 (VAR + TKL + AG) 15’
Scenario 2 30’ Unusual	Run 4	Run 5 (VAR + TKL + SN)

In the solution scenario (Run 2, Run 3, Run 5) controllers operate in a scenario that is comparable to the reference one (Run 1, Run 4), but in the solution scenario the controller is supported by the technical solution.

### Experimental protocols and experiment guidelines

The ethical dimension of the project was assessed using Horizon 2020 Ethics Appraisal Procedure. Based on the ethics assessment performed by Horizon 2020, no ethics issues were identified. However, the experimental protocol was approved by the Project Management Board composed of the following institutions: Austro Control, Croatia Control, DLR-Deutsches Zentrum für Luft- und Raumfahrt, ENAV, University of Bologna. Following the protocols and guidelines defined by the Project Partners in compliance with the requirements defined in the European Union’s grant agreement No. 874470 and in line with the Horizon 2020 ethics guideline, experimental data were collected anonymously in the form of observations, self-assessment questionnaire and non-invasive, objective measurement techniques with no associated risk. During the validation campaign the data were collected in the form of subjective qualitative assessment and objective quantitative measurement.

### Informed consent

All the participants and the institution to which they belong provided informed consent and permitted the release of identifying images for collection and (online) open-access publication. The collection and the processing of personal data were carried out in compliance with the General Data Protection Regulation (GDPR-Regulation EU 2016/679).

## Results

The following section describes the main results obtained through the validation of the proposed concept and relative features (TL, AG, SN). First, the results obtained through objective measurements for the different exercise runs are summarised in Tables 3 and 4. Then, for each one of the three features assessed, the most important objective and Human Performance results are presented in Table 5 and analyzed using bar charts.

**Table 3:** GND ATCO—quantitative data collected during the experiments are shown as average values along with standard deviations (in brackets).

GND ATCO		Total time mean (SD) (s)	Head-up time mean (SD) (s)	Head-down time mean (SD) (s)	No. of switches mean (SD)	No. vocal communications (SD)
TL	Ref	2356 (93)	927.2 (292)	1428.8 (243)	545 (122)	
	Sol	2352.4 (106)	1940.6 (186)	411.8 (231)	308.4 (169)	
AG	Ref	818 (21)	350.6 (79)	467.4 (89)	187 (34)	15.6 (1.74)
	Sol	819 (22)	686.6 (62)	132.4 (67)	73.2 (28)	6.6 (1.36)
SN	Ref	1695.8 (12)	804 (191)	891.8 (184)	365.8 (67)	
	Sol	1717.6 (36)	1300.2 (88)	417.4 (110)	272.2 (81)	

Each solution exercise was repeated by five different participants. The first column reports the total time needed to carry out the exercise, the second and third report the share of time in head-up or head-down position respectively. The fourth column shows the number of switches and the fifth column indicates the number of vocal communication in the exercise regarding the Air Gestures implementation.

**Table 4:** RWY ATCO—quantitative data collected during the experiments are shown as average values along with standard deviations (in brackets).

RWY ATCO		Total time mean (SD) (s)	Head-up time mean (SD) (s)	Head-down time mean (SD) (s)	No. of switches mean (SD)	Time to react to safety event (SD) (s)
TL	Reference	2356.4 (93)	1007.2 (143)	1349.2 (203)	523.2 (138)	
	Solution	2349.4 (108)	1744.2 (182)	605.2 (270)	331.4 (135)	
AG	Reference	818 (22)	393.2 (56)	424.8 (47)	198 (41)	
	Solution	821 (23)	676 (117)	145 (104)	108.8 (60)	
SN	Reference	1694 (17)	809.2 (176)	884.8 (189)	338 (60)	14 (1.50)
	Solution	1721 (32)	1269.4 (273)	451.6 (271)	238.8 (85)	9 (2.10)

Each solution exercise was repeated by five different participants. The first column reports the total time needed to carry out the exercise, the second and third report the share of time in head-up or head-down position respectively. The fourth column shows the number of switches and the fifth column indicates the time to react to the safety event in the exercise regarding the Safety Net implementation.

**Table 5:** Workload, physical workload and situation awareness (SA) respectively assessed through Bedford Scale, 7 points Likert scale and China Lake questionnaires are presented as average values along with standard deviation (in brackets).

		GND+RWY		GND ATCO		RWY ATCO	
		Ref	Sol	Ref	Sol	Ref	Sol
Bedford Scale—avg. workload (SD)	TL	2.6 (1.28)	3.1 (2.07)	2 (0.00)	3.8 (2.71)	3.2 (1.60)	2.4 (0.49)
	SN	2.5 (0.67)	2.3 (0.64)	2.2 (0.75)	2.2 (0.75)	2.8 (0.40)	2.4 (0.49)
Avg. physical workload (SD)	TL	2.3 (1.10)	3.5 (1.36)	1.4 (0.49)	2.8 (1.17)	3.2 (0.75)	4.2 (1.17)
	SN	1.8 (0.75)	3.3 (1.49)	1.4 (0.49)	3.2 (1.60)	2.2 (0.75)	3.4 (1.36)
China Lake—avg. SA (SD)	TL	9.5 (0.67)	7.8 (1.25)	10 (0.00)	7.4 (1.36)	9 (0.63)	8.2 (0.98)
	SN	8.7 (1.00)	8.6 (1.11)	8.8 (1.17)	8.4 (1.36)	8.6 (0.80)	8.8 (0.75)

Each solution exercise was repeated five times by different participants.

### Quantitative objective data

**Figure 10:**
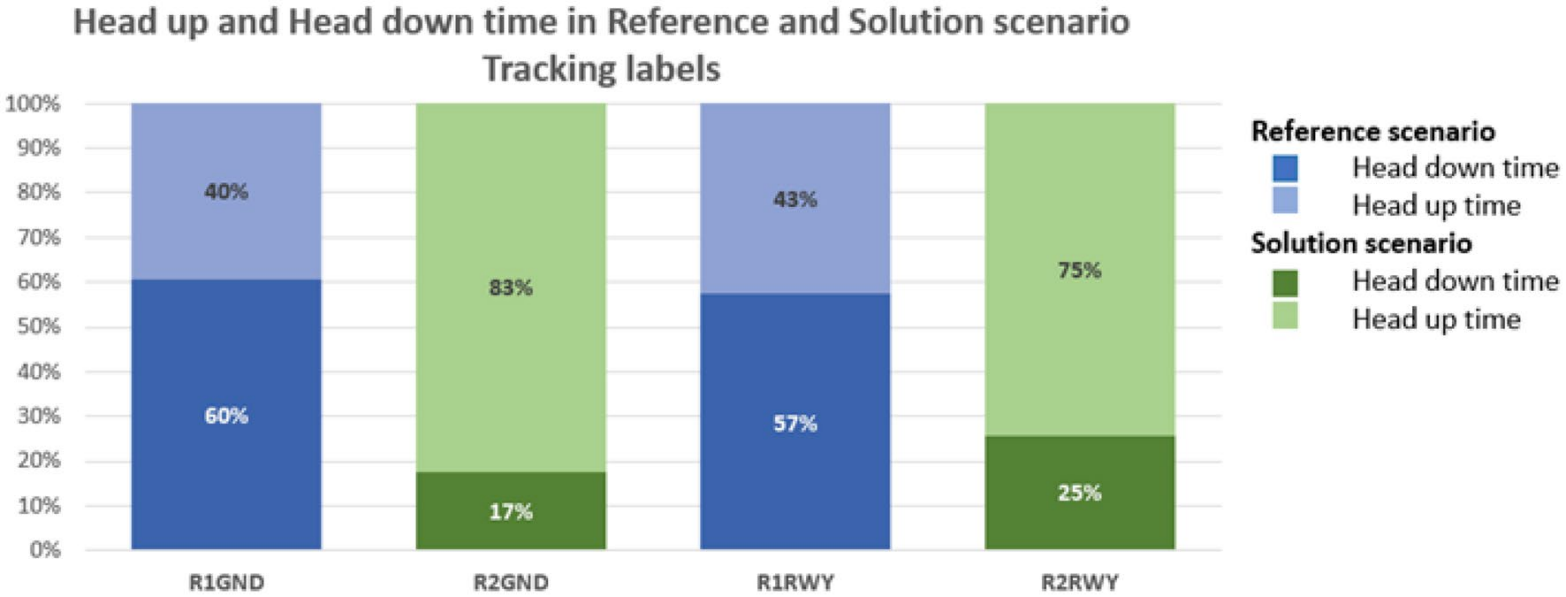
Share of time spent head-down/head-up by the user in Reference and Solution scenario—Tracking Labels exercises. Average values.

**Figure 11:**
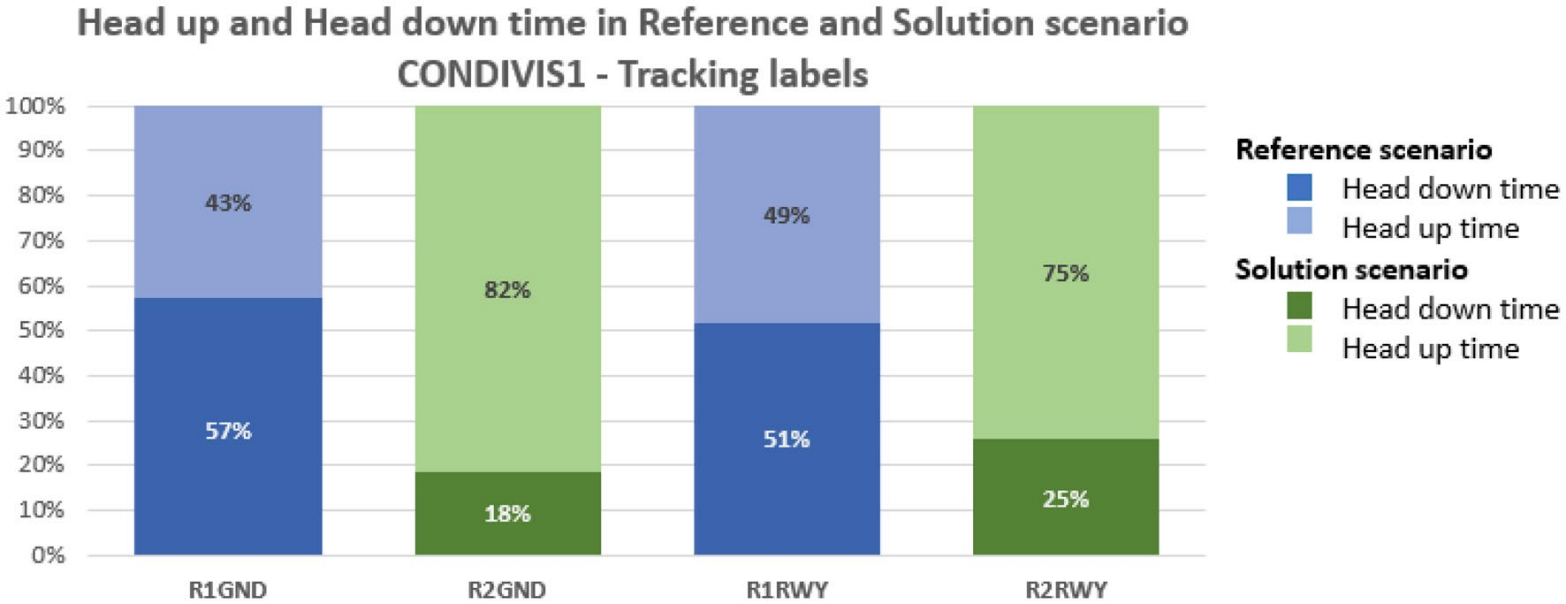
Share of time spent head-down/head-up by the user in Reference and Solution scenario—Tracking Labels exercises in good visibility condition (CONDI VIS 1). Average values.

In the following charts the percentages of head-down/head-up time for the three proposed technical solutions are compared with the reference values. Figure 10 displays the head-down and head-up time of the exercise concerning the introduction of tracking labels for the reference (Run 1) and solution scenarios (Run 2), whilst Fig. 11 displays the head-down and head-up time for the same exercise restricted to the first 15 min of good visibility conditions (CONDI VIS 1).

**Figure 12:**
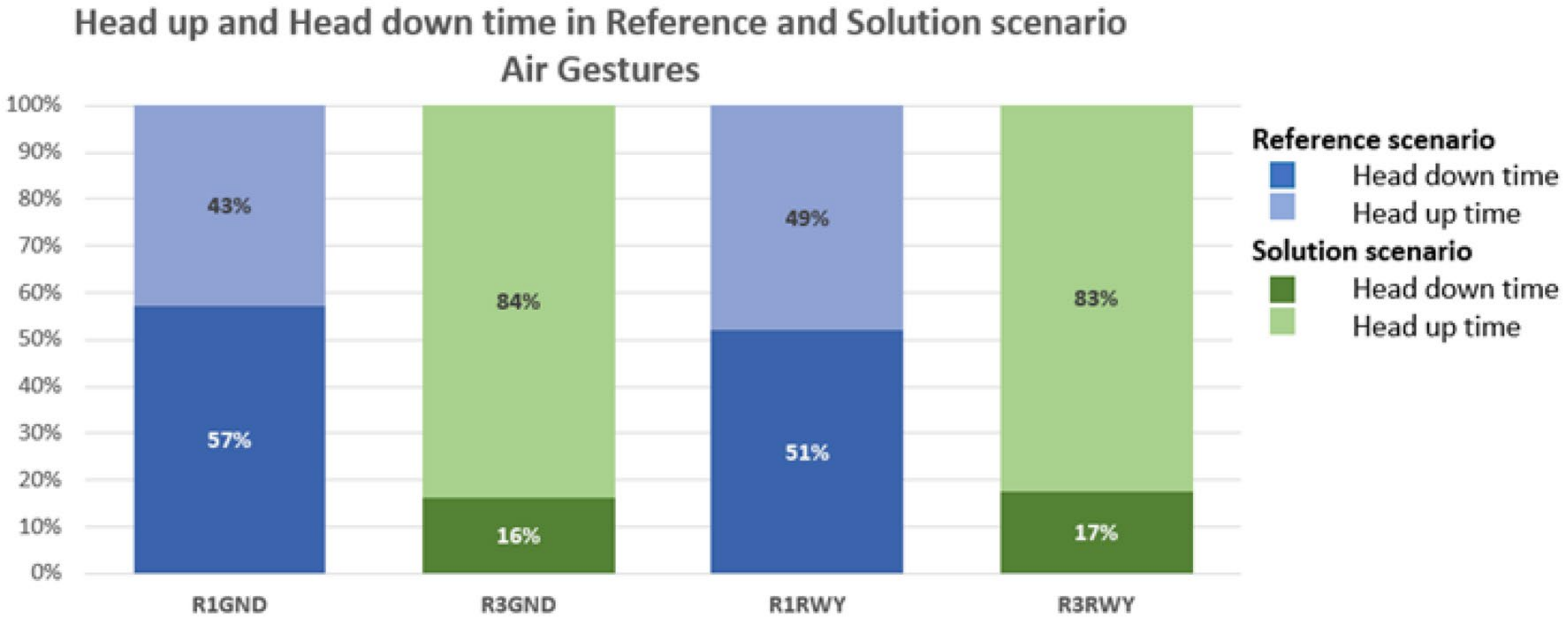
Share of time spent head-down/head-up by the user in Reference and Solution scenario—Air Gestures exercises. Average values.

Figure 12 displays the head-down and head-up times of the exercise concerning the introduction of air gestures for the reference (Run 1-CONDI VIS 1) and solution scenarios (Run 3).

**Figure 13:**
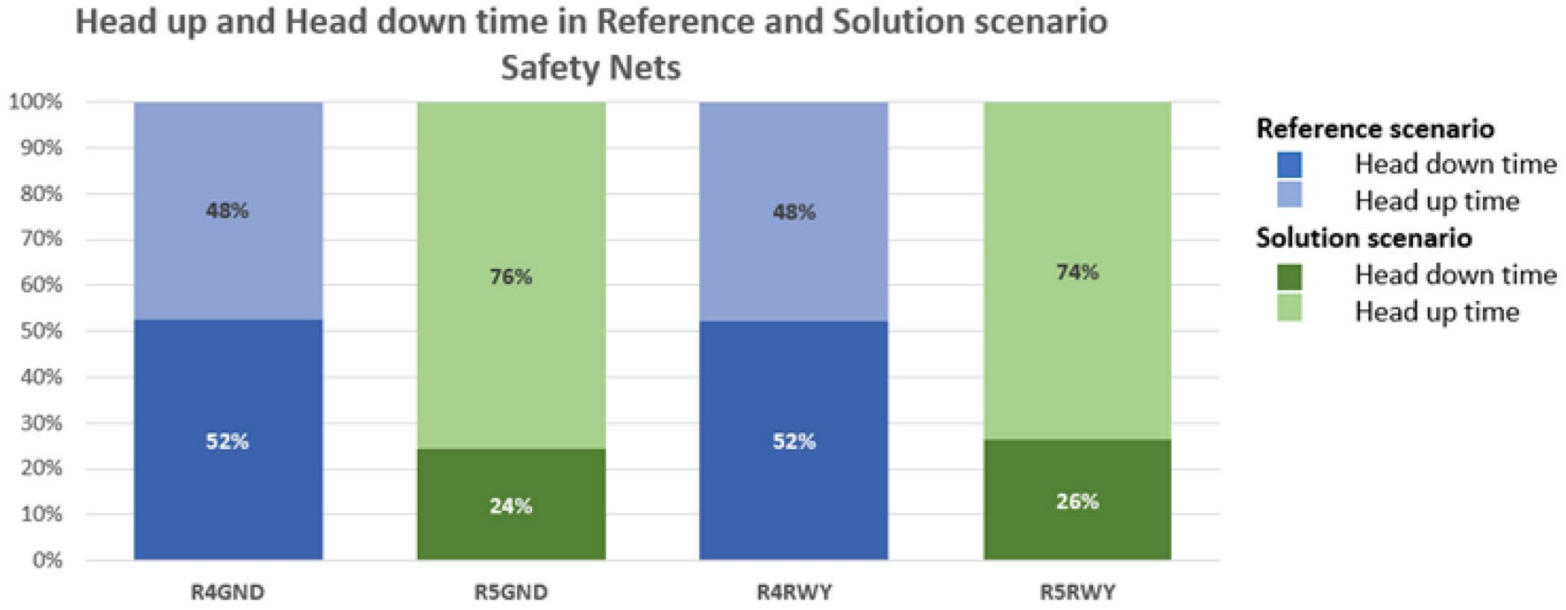
Share of time spent head-down/head-up by the user in Reference and Solution scenario—Safety Nets exercises. Average values.

Figure 13 displays the head-down and head-up times of the exercise concerning the introduction of safety nets for the reference (Run 4) and solution scenario (Run 5).

**Figure 14:**
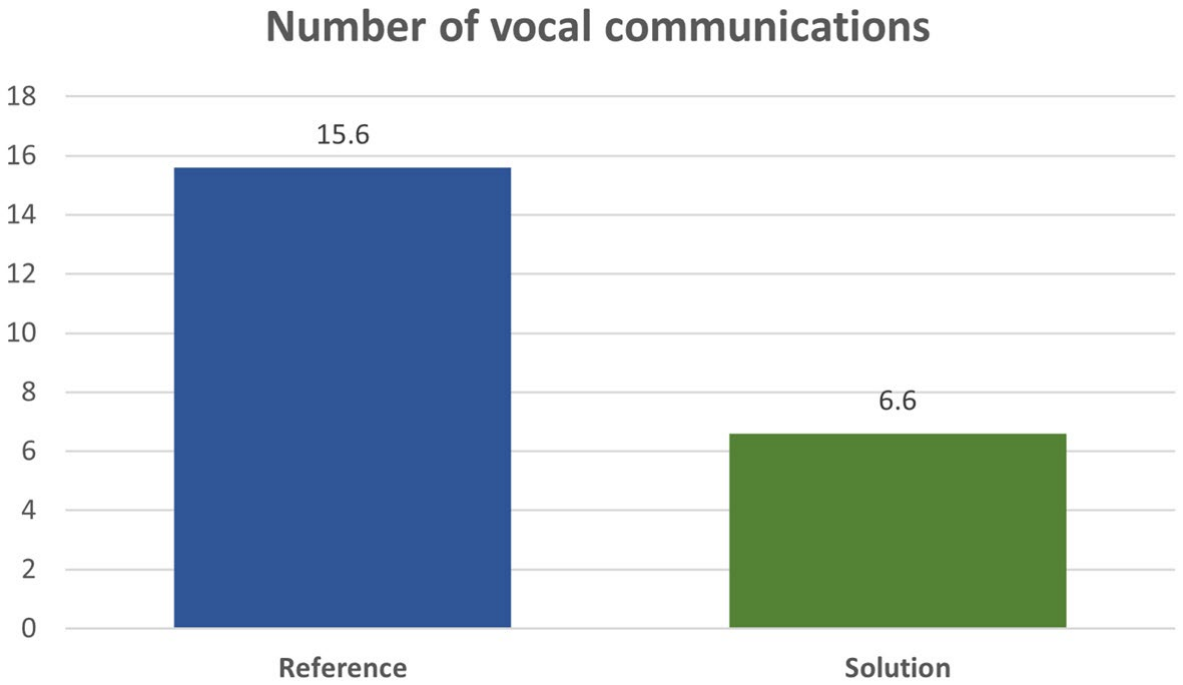
Number of vocal communications in Reference and Solution scenario. Average values.

**Figure 15:**
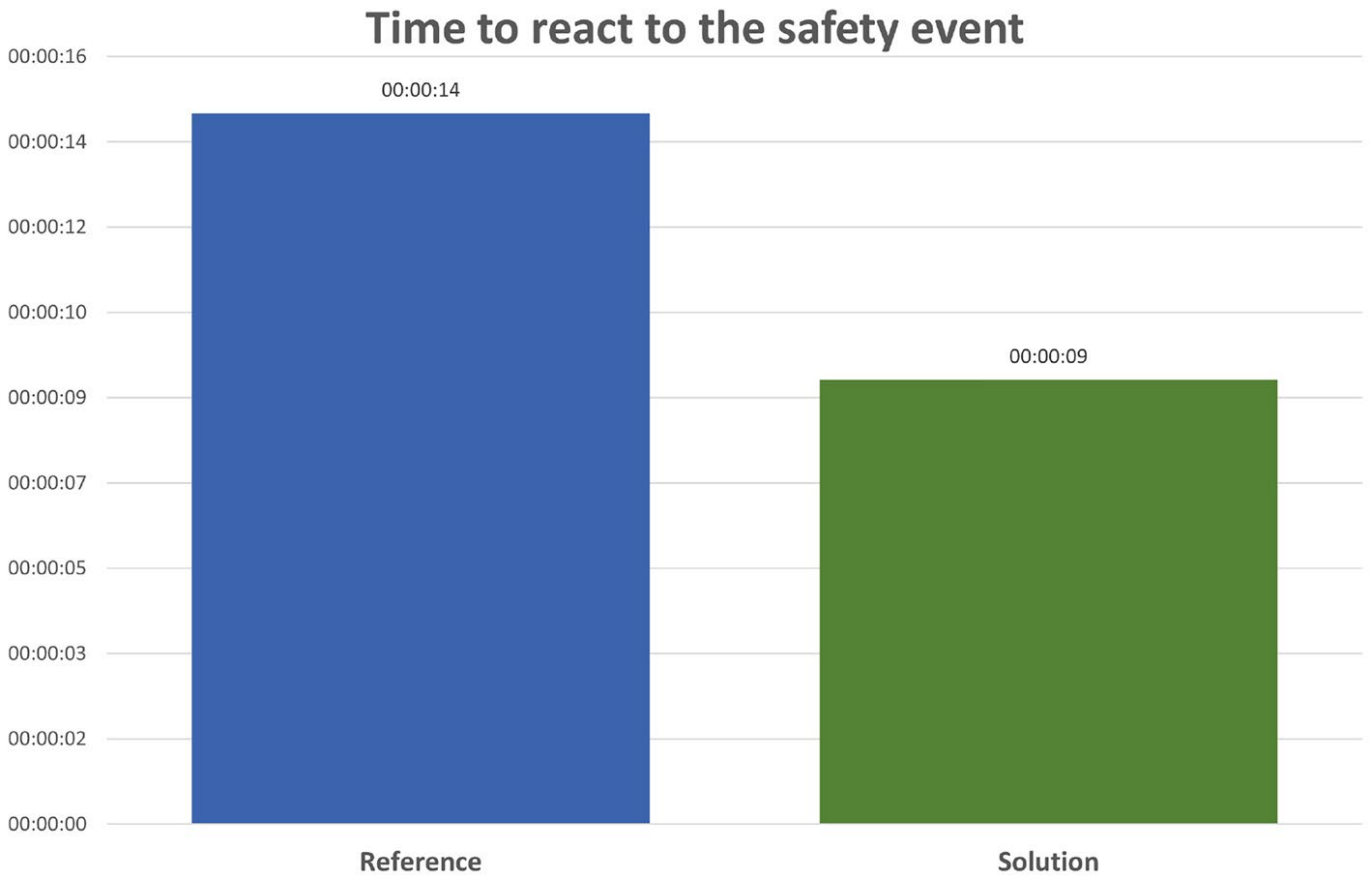
Time to react to a safety event in Reference and Solution scenario. Average values.

Other important results are the number of vocal communications of the GND ATCO (Fig. 14) and the time to notice and react to a safety event of the RWY ATCO (Fig. 15) in the simulations respectively concerning Air Gestures and Safety Net.

### Human performance subjective measurements

**Figure 16:**
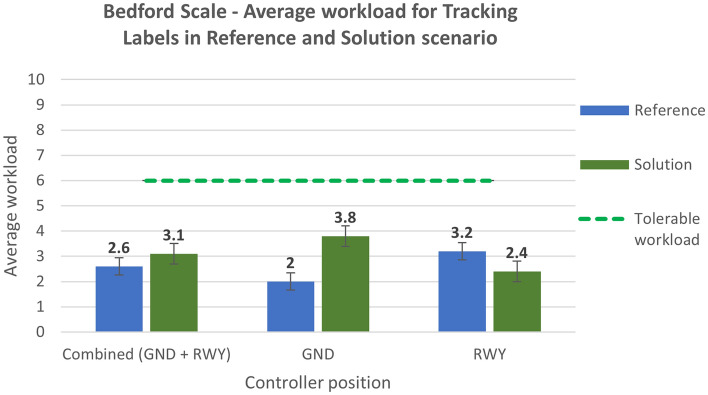
Bedford scale—average workload in Reference and Solution scenario—Tracking Labels exercise.

The Bedford scale, designed to identify an operator’s spare mental capacity while completing a task, was used to estimate the controllers’ workload. Figure 16 compares the average workload experienced by the controllers during the execution of the exercise concerning the TL in the reference (Run 1) and solution scenarios (Run 2).

**Figure 17:**
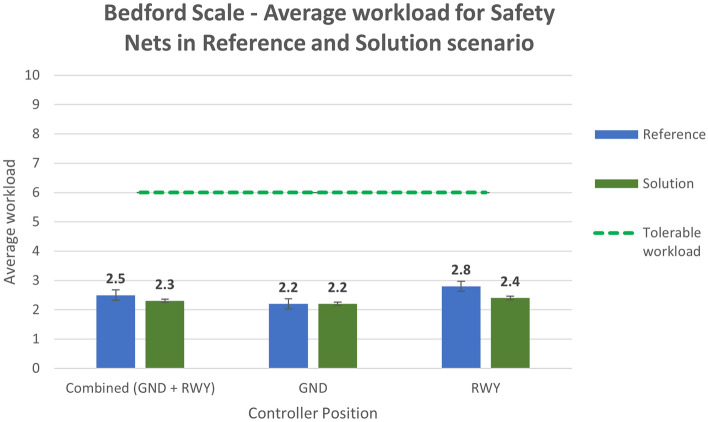
Bedford scale—average workload in Reference and Solution scenario—Safety Nets exercise.

Figure 17 displays the average value of workload for the SN exercise for reference (Run 4) and solution scenario (Run 5).

**Figure 18:**
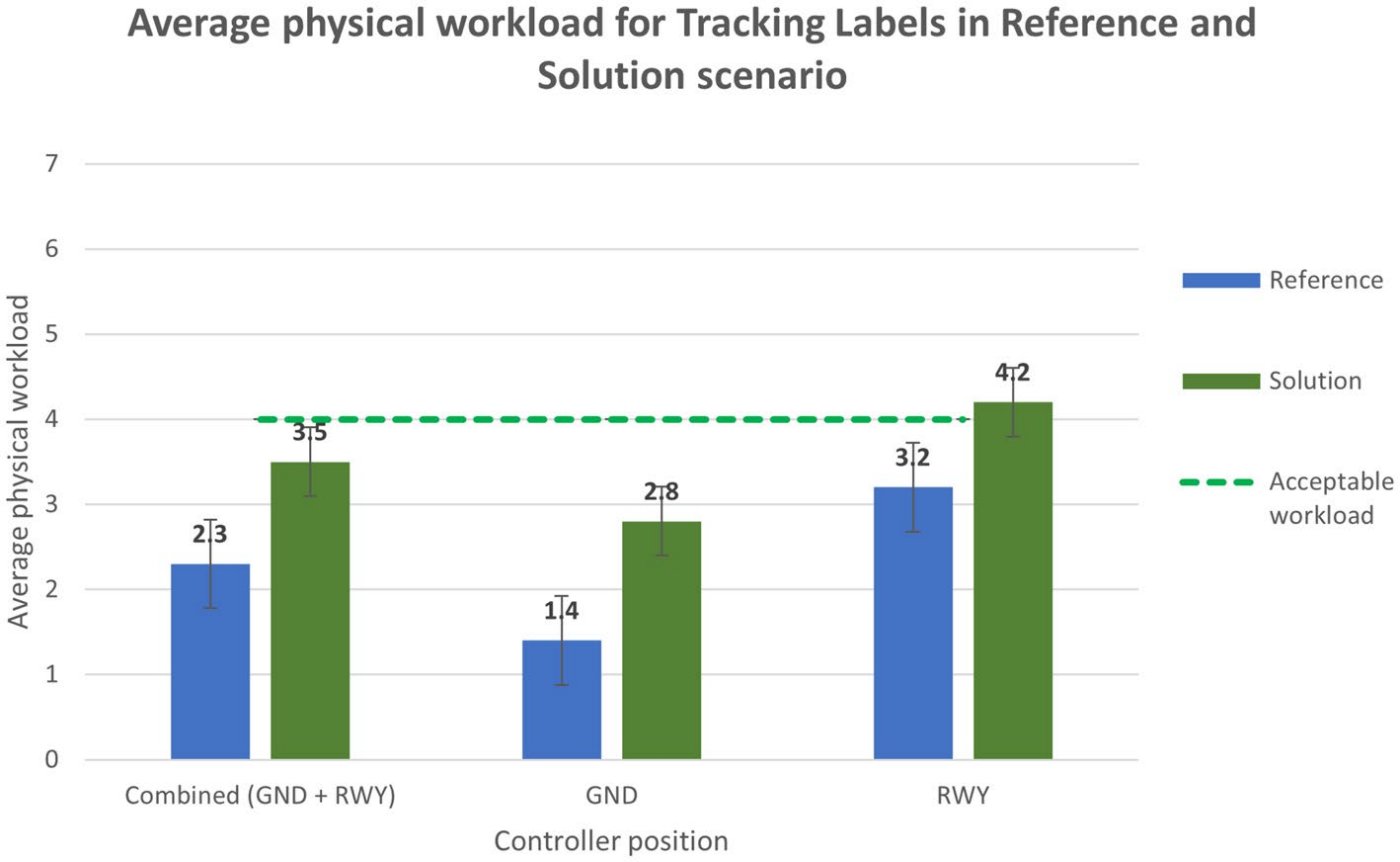
Average physical workload in Reference and Solution scenario—Tracking Labels exercise.

**Figure 19:**
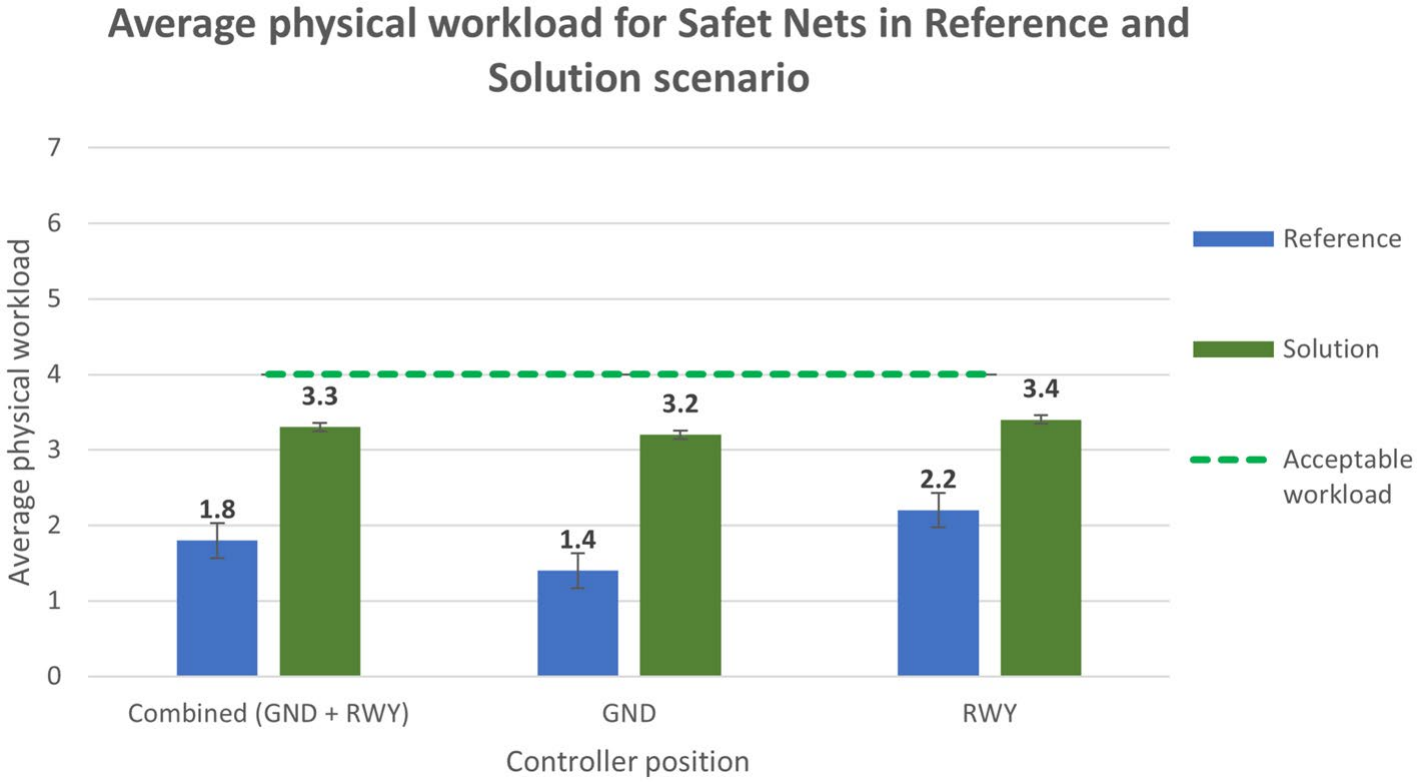
Average physical workload in Reference and Solution scenario—Safety Nets exercise.

The physical workload is measured using a 7 points Likert scale for the reference scenarios, the tracking label (Fig. 18) and the safety net (Fig. 19) solutions and the working positions experimented with.

**Figure 20:**
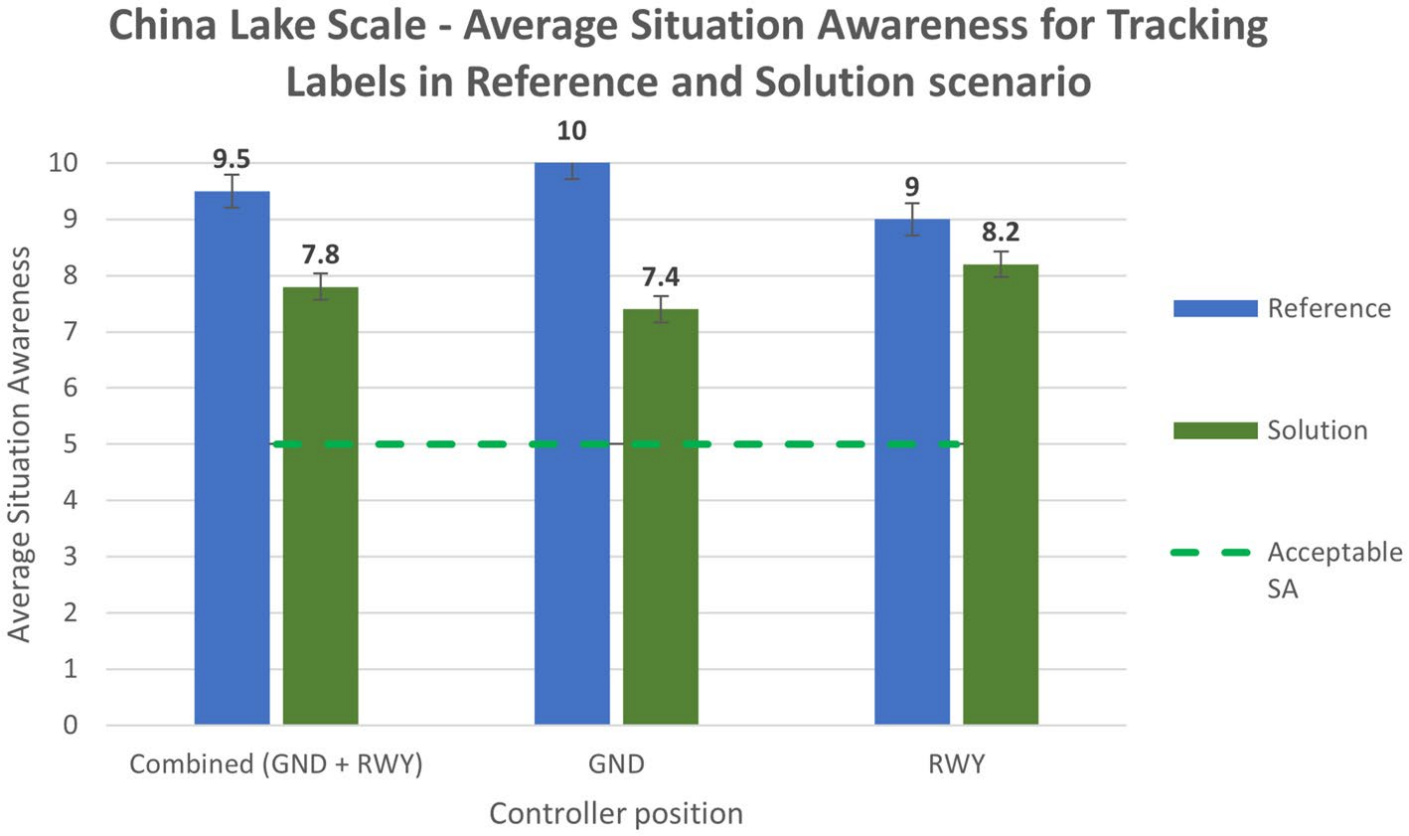
China Lake scale—average situation awareness in Reference and Solution scenario—Tracking Labels exercise.

**Figure 21:**
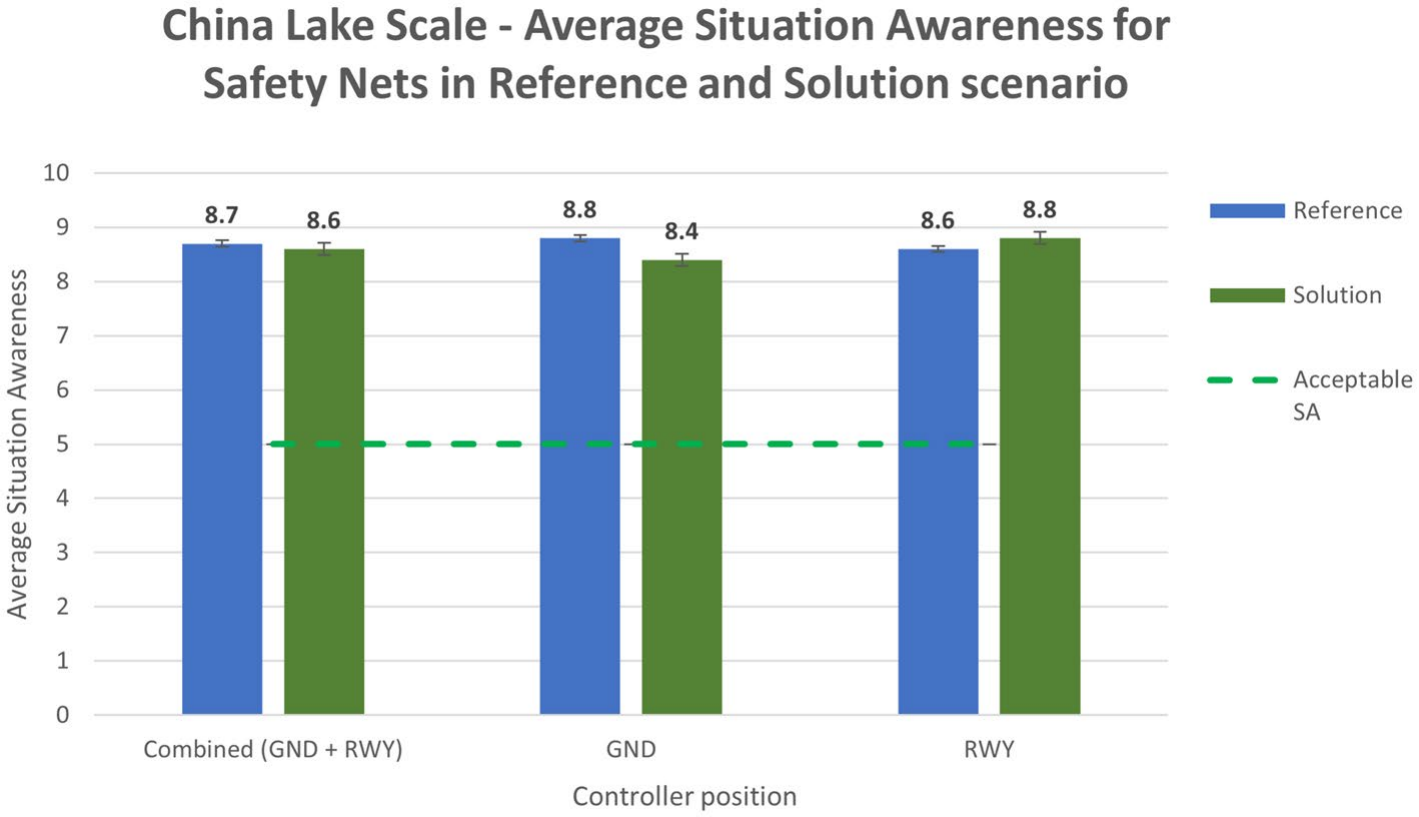
China Lake scale—average situation awareness in Reference and Solution scenario—Safety Nets exercise.

The situation awareness was analyzed using the China Lake questionnaire. The average values for the reference and solution scenarios are reported in the following bar charts (Figs. 20 and 21).

**Figure 22:**
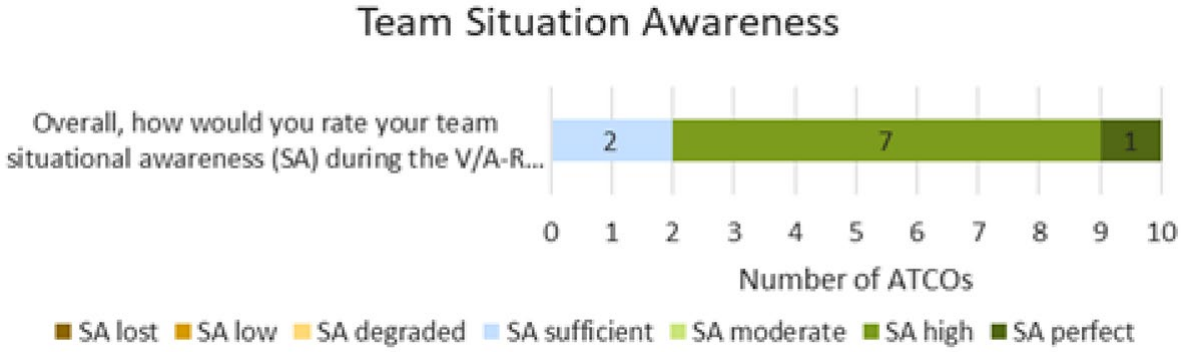
Team situation awareness in solution scenario—Tracking Labels and Safety Nets exercises.

Team Situation Awareness is measured in a 7 points Likert scale as reported in Fig. 22.

**Figure 23:**
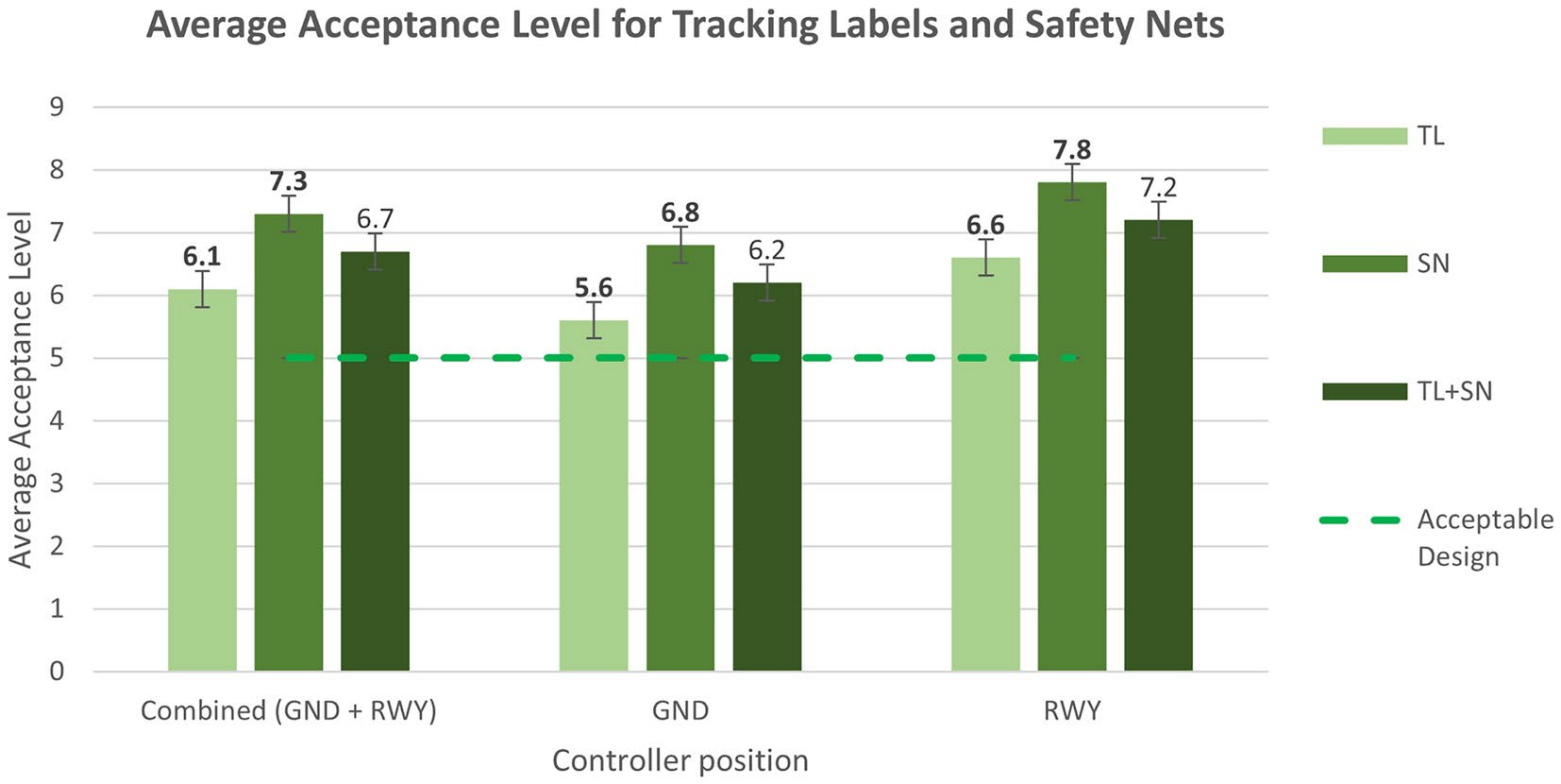
CARS scale—average acceptance level in Solution scenario—Tracking Labels and Safety Nets exercises.

The CARS scale results for acceptance are reported below (Fig. 23).

## Discussion

Assessing the results of objective quantitative and subjective qualitative data, the proposed solutions prove to have an overall positive effect on human performance and efficiency for both working positions, namely Ground and Runway controllers. Compared to the reference scenario, all the three technical solutions (TL, AG, SN) provide a substantial increase in the time spent in head-up position looking at the out-of-the-tower environment rather than at the HDE (Fig. 10, 11, 12 and 13). The data concerning head-down and head-up time are strictly related to the number of switches between the two positions. As can be observed in Tables 3 and 4, with the introduction of the HMD device, the number of switches is significantly reduced. This reduction is particularly relevant since the continuous change of perspective on the same out-of-the-tower environment would lead to a decrease in situational awareness^1^.

Moreover, when dealing with the simulation exercise regarding the introduction of Air Gestures, an important parameter to evaluate is the number of vocal communications exchanged by the GND ATCO. The possibility to interact with the pseudo-pilot, and in particular to deliver some clearances, through gestures can lead to a reduction in the number of vocal communications. The average reduction of this number between the reference and solution scenario is about 56% (Fig. 14), and as some controllers pointed out it also reduced the associated risk of miscommunication.

Lastly, assessing the introduction of Safety Nets it can be observed that the time needed by the RWY ATCO to notice and react to a safety event is reduced by almost 65% in the solution scenario with respect to the reference scenario (Fig. 15).

Analysing the subjective data, the results show a positive assessment of the HP key indicators. Results related to the workload show that the average workload is always well below the acceptable threshold for both the Ground and the Runway controller working position in all the solution runs (Figs. 16 and 17). According to these data, the working position which benefits the most from the introduction of the technical solution is the Runway. The simulation results do not have the same trend for the physical workload, mainly due to the wearable device that, despite being reported as acceptable by the controllers, has in any case an influence on the perceived physical workload. This effect is shown in Figs. 18 and 19 detailing the average values of the physical workload on a 7 point Likert scale for the reference scenarios, the tracking labels and safety nets solutions and the working position experimented with. The situation awareness was analyzed through the China Lake questionnaire. As reported in Figs. 20 and 21 the ATCOs always had a good mental picture even if a slight decrease was observed in the solutions scenarios especially for the Ground controller working position. Indeed, the ATCOs suggested different improvements in the information provided in the head-mounted display in terms of size, colours, background and positions of the labels which in some phases of the flight overlapped covering the scene.

On the other side, the team situation awareness (Fig. 22) was always at sufficient level and ATCOs’ subjective feedback was that team situation awareness could even be improved considering that both ATCOs have additional information in Head-up for all the flights, possibly reducing the need of coordination between the two controller positions.

Acceptance, measured using the CARS standard scale (Fig. 23), trust and job satisfaction were also rated at an acceptable level for the tracking labels and safety nets for both the working positions.

Considering the lower level of maturity of the Air Gestures solution, initial qualitative feedback were only collected to feed the next design phase and thus no standard questionnaires are provided. As a whole, the concept presented of multimodal interaction encompassing gestures and voice received positive feedback from all the users, however some usability improvements will be considered to achieve a higher level of maturity of the solution. Indeed the majority of ATCOs had difficulties using Air Gestures due to a low gesture recognition rate. These difficulties could be mitigated to the point of being totally eliminated through a specific training, but in this phase of the validations they have negatively impacted the human performance of the GND ATCOs. Indeed, the ATCOs mentioned a possible impact on their physical workload, situation awareness, and the perceived potential for human error.

Even considering these downsides, no impact on the perceived potential for Human Error was reported by the users. On the contrary, 80% of the controllers (4) observed that the VR/AR Air Gestures have no or a positive impact on the trust level and on Acceptance and Job satisfaction level.

## Conclusions

This paper introduces the concept of an innovative Augmented Reality HMI for airport control towers and describes the exercises conducted to validate the technical solutions developed, focusing on the simulation platform and exploited technologies to demonstrate how Virtual and Augmented Reality, along with Tracking Labels, Air Gestures and Safety Nets, enable a more natural and effective interaction in the control tower, on the one hand improving the performance and on the other the situational awareness of the Air Traffic Control Operators.

As expected, the results show that the prototype concept developed and implemented on the UNIBO platform is feasible from both an operational and technical perspective. The solution proposed proves to support the ATCO in working in a head-up position more than head-down even with low-visibility operational scenarios, and to lower the time to react in critical or alerting situations.

The positive impact of the HMI implementation on human performance metrics, as evidenced by feedback from ATCOs, underscores its potential to mitigate workload, reduce the potential for human error, and enhance trust, acceptance, job satisfaction, and perceived safety. However, several limitations and challenges must be addressed for its full potential to be realised. In particular, the following limitations and challenges have emerged in developing this study:

- synthetic field of view constraints: the current implementation is constrained by the limited field of view of the AR device, potentially hindering the ATCOs’ awareness of peripheral information crucial for decision-making.
- Tracking Label design and positioning: while TL augment object identification, their design and positioning may require refinement to better align with user preferences and operational needs.
- Cognitive load and workflow impact: the introduction of additional overlays raises concerns about cognitive load and its potential negative impact on workflow efficiency. Balancing information richness with cognitive load remains a key challenge.
- Transition to Real-World scenarios: while successful in simulated environments, transitioning to real-world scenarios presents unique challenges, including regulatory compliance, infrastructure integration, and user acceptance.

To assess these limitations, future developments should focus on:

- Enhanced synthetic field of view: addressing the limitation of the AR device’s field of view by exploring technologies such as wide-angle lenses or panoramic displays could provide a more comprehensive situational awareness.
- User-Centric Design iterations: continuous iteration based on user feedback and ergonomic studies is essential to refining the HMI’s design, ensuring its alignments with user preferences and operational requirements.
- Dynamic information prioritisation: integrating AI-driven logic systems to dynamically select and prioritise displayed information can mitigate cognitive overload while ensuring relevant data is readily available to ATCOs.
- Attention guidance and predictive operations: incorporating attention guidance features, coupled with AI, can enable a transition from reactive to predictive ATC operations, fostering proactive risk mitigation and enhancing overall airport safety.
- Real-World validation: future research should prioritise real-world validation in operational airport control towers to assess the scalability, reliability, and user acceptance of the proposed HMI solution.
- Advanced Air Mobility (AAM) integration: in implementing new solutions for the control tower, future tasks which could be assigned to the controllers should be considered, with a special focus on the role of AAM which is now being researched and will probably require the integration of urban and Unmanned Aerial System traffic in the traffic management scenario in the next decade.

By properly addressing these limitations and future developments, the proposed concept has the potential to lead to a significant benefit for the future aviation system, including, but not limited to financial savings for carriers and Air Navigation Service Providers, increased safety for passengers and improved resilience and efficacy for the control tower IT systems.

## Data Availability

The datasets used and/or analysed during the current study are available from the corresponding author on reasonable request.
